# Cyberbullying and Adolescent Neurobiology

**DOI:** 10.3389/fpsyg.2020.01511

**Published:** 2020-06-26

**Authors:** Larisa T. McLoughlin, Jim Lagopoulos, Daniel F. Hermens

**Affiliations:** Thompson Institute, University of the Sunshine Coast, Birtinya, QLD, Australia

**Keywords:** cyberbullying, brain development, adolescence, literature, mental health, neurobiology

## Abstract

Whilst it is well documented that cyberbullying is linked to poor mental health outcomes, limited research has examined how cyberbullying may influence brain development adolescents, and the influence of each of these factors. The article’s primary objective was to develop an understanding of research to date that addresses any relationship between adolescent brain development and cyberbullying. The current article reviews any existing literature regarding the impact of cyberbullying on adolescent brain development, paying particular attention to research using magnetic resonance imaging (MRI) techniques. Whilst brain studies have examined neural mechanisms associated with conduct disorders, antisocial behavior, and aggression in young people; there is a paucity of research examining these factors specifically in relation to cyberbullying. In particular, little research has examined how MRI research could help understand how the brain is affected by cyberbullying, not only in bullies and victims but also bystanders. This article highlights the gaps in the cyberbullying field in relation to neuroscience research, and the need for further, longitudinal research examining cyberbullying and how it may affect brain development in young people. This article concludes by suggesting a framework for future research, and highlights the importance of future findings for developing interventions and understanding short and long term effects.

## Introduction

There has been substantial research demonstrating the dynamic (both linear and non-linear) changes in gray matter (GM) and white matter (WM) that occur during the adolescent period. However, research regarding how such brain changes may be influenced by experiences of cyberbullying has received little attention. Cyberbullying, is defined as an aggressive, repeated, intentional act carried out on an individual via electronic forms (Smith et al., 2008), and can have serious adverse mental health outcomes (Campbell et al., 2012; van Geel et al., 2014; Spears et al., 2015; Fahy et al., 2016; Kim et al., 2016; Le et al., 2017; McLoughlin et al., 2018, 2019).

Across studies, females are more likely to be cybervictims and male are more likely to cyberbully (Li, 2006; Cross et al., 2009; Sakellariou et al., 2012; Hemphill and Heerde, 2014). In addition, the Health Behavior in School-Aged Children (HBSC) international report highlighted that bullying victimization declined between ages 11 and 15 years, whereas bullying perpetration significantly increased between ages 11 and 15, and that this differs between genders (Currie et al., 2009). In contrast, Zych et al. (2015) in their review suggest that the relationships between age, gender, and involvement in bullying and cyberbullying are complex, and that across studies these relationships may, in fact, be weak. Evidently, these varied findings highlight the need for longitudinal research to examine patterns in cyberbullying over time, specifically in the context of age, gender, and neurobiological investigations.

Research examining neurobiological changes during adolescence, suggests that a high level of brain plasticity characterizes early childhood and adolescent stages of development (Bradshaw et al., 2012), and as such, this is an optimal time for learning and development. Synaptic formation peaks around 12 years of age, followed by a general “pruning” of surplus or underused synapses. Furthermore, adolescents go through significant emotional, hormonal, and behavioral changes, with a heightened responsiveness of the brain’s socio-emotional system, which typically affects the capacities of their still maturing self-regulatory system (Steinberg, 2013).

Numerous studies have shown an increase in WM and decrease in GM density in the frontal and parietal cortices throughout adolescence (Pfefferbaum et al., 1994; Giedd et al., 1996, 1999; Reiss et al., 1996; Sowell et al., 2001, 2003; Barnea-Goraly et al., 2005; Blakemore and Choudhury, 2006; Giedd, 2008; Ostby et al., 2009) and that these changes may aid in identifying core neurobiological characteristics associated with the onset of mental illness (Hatton et al., 2012; Lagopoulos et al., 2012, 2013). More specifically, Ostby et al. (2009) found in a study of 171 children and young adults (aged 8–30 years) that while GM decreased non-linearly in the cerebral cortex and linearly in the caudate, putamen, pallidum, accumbens, and cerebellum, the amygdala and hippocampus showed slight, non-linear increases in volume. Critically, Mills et al. (2014) explains that the development of the “social brain” regions (prefrontal cortex, temporoparietal junction, posterior superior temporal sulcus, and anterior temporal cortex) during the adolescent years is important for social understanding and communication, and hence plays a vital role in social issues such as cyberbullying.

Also, WM increases non-linearly within the cerebrum and cerebellum, with an earlier maturation in cerebellar WM (Ostby et al., 2009). Furthermore, Mills et al. (2016) suggest that that WM volume increases until between the ages of 10–15 years, then decreases again until the early twenties where it then stabilizes. This development is particularly important, as WM pathways play a key role in cognitive, behavioral, emotional and motor development during childhood and adolescence, and may explain why adolescents generally are less psychosocially mature than adults; suggesting a key role for WM development in the context of cyberbullying during adolescence (Corrado and Mathesius, 2014).

Adolescents also experience significant changes in functional and structural connectivity and integrative processing, with very important changes in the balance between limbic/subcortical and frontal lobe function (whereby the latter takes control of the former). Collectively, the dynamics and significant changes through adolescent brain development influence the notable changes in cognition, emotion, and behavior (Giedd, 2008). Furthermore, there may be an association between hippocampal volumes and psychological distress in adolescence which may play an important role in the emergence of mental illness (Broadhouse et al., 2019). Finally, due to the many changes occurring in the brain as adolescents mature, they are at a heightened vulnerability to problems affecting regulation of mood and behavior, and are therefore more prone to risk-taking, recklessness, and the onset of emotional and behavioral problems (Steinberg, 2005; Casey et al., 2010).

Rates of depression and anxiety increase as children enter adolescence, and is a peak period in terms of onset of many major mental disorders (Paus et al., 2008), suggesting that this transition is a vulnerable time for young people’s mental health (Hankin et al., 1998). This is of particular relevance as adolescents experience a reorientation toward peers and away from their parents, and consequently may experience heightened stress if rejected by their peers, which can increase the onset of mood disorders (Masten et al., 2011). Masten et al. (2009, 2011) found that adolescents with increased activity in the subgenual anterior cingulate cortex (ACC) in response to peer rejection, were more likely to develop depressive symptoms and experienced greater distress, suggesting that the degree of neural activity displayed by adolescents in response to social rejection may relate to their emotional sensitivity to such an event. Given the significant brain changes that are occurring during adolescence, there are links between this time of major transitions, socialization, and bullying issues (Steinberg, 2005).

The aim of this article was to review the literature that has explored relationship between neurobiology and cyberbullying in adolescents in some way. Addressing gaps in this area of research is important, as findings may lead to a better understanding of cyberbullying which may identify potential causative factors as well as facilitate the identification of appropriate treatments and interventions. Thus, in this mini review, we pose the following questions: (i) are there specific factors unique to adolescent neurobiology which predisposes individuals or increases their risk to adverse reactions to cyberbullying behavior? (ii) To what extent does cyberbullying influence an adolescent’s neurobiology?

## Cyberbullying and the Brain

Whilst the negative outcomes associated with cyberbullying are well documented, research investigating the relationships between neurobiology and the adolescent brain, cognition, and cyberbullying is lacking. Research in this area is important, as Lamblin et al. (2017) state that genetic influences on brain structure and function impact the quality and quantity of social ties during adolescence, and that the brain and social environment sculpt each other throughout adolescence and can increase risk or promote resilience for mental illness.

To our knowledge only nine studies have specifically addressed neurobiology, the brain, and cyberbullying or traditional bullying/social media use (Table 1). González-Cabrera et al. (2017) found that patterns of cortisol release and perceived stress in 11–18 year old’s are related to cyberbullying roles, with cybervictims and cyberbully victims exhibiting higher cortisol secretion levels and greater perceived stress, as compared to cyberbullies and cyberbystanders. Furthermore, the lowest cortisol secretion was observed in serious cyberbullies (González-Cabrera et al., 2017). Similarly, du Plessis et al. (2019) found that cortisol moderated the relationship between traditional bullying childhood victimization and adolescent ventrolateral prefrontal cortex (vlPFC) structure, and that this was dependent on gender. That is, boys with higher experiences of childhood victimization showed high cortisol levels and a smaller vlPFC structure, compared to those with low cortisol and low victimization. The researchers suggested that this may be due to a stress sensitivity that could influence brain development, especially in boys, and that victimization could be one of these stressors that could have an impact on the brain. Whilst both these studies suggest that there are biological markers associated with different roles in cyberbullying, and that cortisol levels could be particularly important in regard to brain development during adolescence, further research is needed in order to understand how this may influence adolescent brain development over time. The study by du Plessis et al. (2019) also focused on traditional bullying rather than cyberbullying.

**TABLE 1:** Studies that specifically addressed neurobiology, the brain, and cyberbullying or traditional bullying/social media use.

References	Focus	Cyberbullying data
Vaillancourt et al., 2013	The biological underpinnings of peer victimization	No – traditional bullying
Sherman et al., 2016	Effects of peer influence on neural and behavioral responses to social media	No – social media use
González-Cabrera et al., 2017	Relationship between cyberbullying roles, cortisol secretion and stress	Yes – cyberbullying data
Crone and Konijn, 2018	Media use and brain development during adolescence	No - review
Quinlan et al., 2018	Peer victimization, adolescent brain development and psychopathology	No – peer victimization
Muetzel et al., 2019	Frequent bullying involvement and brain morphology in children	No – traditional bullying
du Plessis et al., 2019	Longitudinal study on childhood peer victimization and the brain	No – traditional bullying
McLoughlin et al., 2020b	A pilot functional magnetic resonance imaging study on cyberbullying	Yes – cyberbullying data
McLoughlin et al., 2020a	Hypothesis/protocol paper on cyberbullying and neuroimaging	No – protocol

In a review of adolescent brain development, Sherman et al. (2016) reported that online social interactions are associated with similar structural correlates and patterns of brain activity to those observed in the context of real-world relationships. Moreover, young people respond in a similar manner to positive feedback online (such as “likes” on their photos or updates) as they would in a face-to-face conversation (Sherman et al., 2016). More specifically, when adolescents viewed photos with many (compared with few) likes, greater activity in neural regions responsible for reward processing, social cognition, imitation, and attention were activated. Whilst these findings do shed some light on how the brain may respond to online interaction, these findings are not focused on cyberbullying interactions, and are cross sectional. A study which mimics social media conditions would be ideal to understand how the brain responds to cyberbullying stimuli.

Recently, our group (McLoughlin et al., 2020a, b) has addressed this gap, and was the first to evaluate cyberbullying scenarios using neuroimaging. We developed a protocol for using functional magnetic resonance imaging (fMRI) to measure how cyberbystanders respond to cyberbullying stimuli (McLoughlin et al., 2020a), and found that viewing such stimuli activated responses across the many regions of the brain, including those linked to social and emotional processing (McLoughlin et al., 2020b). We also found that those with no prior experience of cyberbullying showed a greater response in the area of the brain responsible for feeling self-conscious (McLoughlin et al., 2020b). In addition, we found that females had a greater response in the right ACC, which is the brain region that plays a key role in the processing of empathy when witnessing cyberbullying. This highlights that there may be significant differences in how the brain is affected by cyberbullying between males and females. However, this study was a pilot, and therefore involved a small sample of cross-sectional data, and as such longitudinal research is needed to better explicate this. No research has addressed how age may influence the way the brain responds to cyberbullying, but it would be a worthwhile line of future research.

In addition, an area lacking research is the influence of mental health problems on any association between cyberbullying involvement and brain development in adolescence. Links between adolescent brain development, peer victimization and psychopathology has been investigated by Quinlan et al. (2018). Whilst not focused specifically on bullying, the researchers did find that changes in left putamen volume were negatively associated with generalized anxiety, and peer victimization was indirectly associated with generalized anxiety via decreases in putamen volume. The authors suggest that these results could indicate that victimization during adolescence could lead to psychopathology-relevant deviations from normative brain development. This area needs more attention in order to understand the influence mental health problems may have on any association between cyberbullying involvement and brain function, and future studies should investigate this. In particular, longitudinal studies should be undertaken to help understand this.

A recent thorough review on media use and brain development highlighted that neuroscience is of vital importance in the future in terms of understanding the developmental sensitivities related to adolescents’ media use over time (Crone and Konijn, 2018). The authors argue that adolescents are particularly sensitive to acceptance and rejection, and that social media exacerbates this, making adolescents vulnerable to emotional sensitivity and poor cognitive control (Crone and Konijn, 2018). In addition, a review on traditional bullying in young people found that the brain experiences peer victimization in a similar way to physical pain, and that these experiences can become biologically embedded in the physiology of the developing person, thereby increasing their risk of developing mental health problems (Vaillancourt et al., 2013). Recently, Muetzel et al. (2019) conducted a study of 2,602 children regarding traditionally bullying, and involved the 8-year-old children, their parents and teachers reporting on common forms of child bullying involvement (physical, verbal, and relational), and then completing a structural MRI scans when the children were 10 years old. The study found that those children who were frequently bullied had thicker cortex in the fusiform gyrus, a region suggested to be implicated in a wide array of functions, including facial and emotion processing, language, and theory of mind (Muetzel et al., 2019). Whilst these aforementioned studies shed light on how victims of bullying perceive their bullies and highlights that frequent bullying could affect brain development, further research is needed which focuses on cyberbullying specifically, as well as over time. Studies which involve repeated MRI scans across adolescent would be ideal, in order to fully understand how cyberbullying experiences at different stages of adolescent (or throughout) influences brain development.

## Conclusion and Future Direction

It is well documented that cyberbullying can lead to negative mental health outcomes, however, research examining how this relates to brain development and neurobiology during adolescence has received little attention, and yet is of considerable importance.

Most research into cyberbullying has relied heavily on self-report. Whilst this is an essential part of gathering information about cyberbullying experiences, research that includes additional measures, such as brain imaging and cognitive assessments, will go beyond subjective information and will enable researchers to better understand adolescent cyberbullying and how experiences influence the development of relationships, cognition and neurobiology (George and Odgers, 2015). Furthermore, this information could highlight opportunities for neuroscience to identify the potential of the adolescent brain, and inform opportunities for adolescents to thrive in different developmental stages (Johnson et al., 2009). Indeed, Smith and Jones (2012) proposed that developmental cognitive neuroscience could help to better understand the factors that might make a child vulnerable to becoming a bully or a victim, as well as aid in developing tailored interventions.

The aforementioned studies have primarily been cross-sectional, and these highlight the need for longitudinal research to understand factors such as vulnerability and changes over time. Specifically, longitudinal studies employing brain imaging and cognitive assessments in conjunction with measures of cyberbullying as well as traditional metrics of mental health (e.g., psychological distress) would be extremely valuable. Research such as this could inform interventions for both cyberbullies and cybervictims, and improve behavioral, social, and academic outcomes. This research also could inform neurodevelopmentally sensitive preventive interventions which target cyberbullying behavior (Bradshaw et al., 2012). Furthermore, whilst it is recognized that there would be important differences in the brain responses to cyberbullying that are due to differences in age and gender, there is little research to date that has specifically addressed this. Indeed, this review has discussed the important stages of change the adolescent brain goes through, therefore, it would be expected that the brain may be influenced by cyberbullying differently according to age and the associated maturational processes. Future research should address this.

In addition, given the lack of research available addressing cyberbullying, much of the theories discussed in this article are based on traditional bullying and the associated neurobiology. Thus, future studies need to explicitly compare both traditional bullying and cyberbullying, however, given the overlap between the two forms of bullying, it is likely that the relationship between cyberbullying, cognition, and the brain will be similar to those findings discussed regarding traditional bullying, and vice versa. That being said, longitudinal studies could identify if cyberbullying has harsher or additional negative effects on the brain, especially given its fast, widespread, and repetitive nature.

### Implications and Proposal Working Forward

**FIGURE 1:**
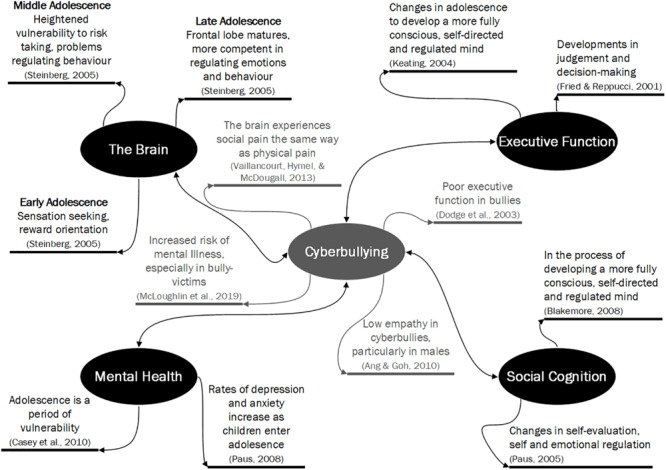
Changes in the brain and cognition occurring during adolescence which may influence cyberbullying behaviors. Black text represents changes and patterns to the brain and to cognition that occur during adolescence. The gray text represents aspects of cyberbullying associated with each of these four major factors discussed throughout this article. Furthermore, the associations between each factor and cyberbullying are represented as bi-directional in the figure, however, further research (in particular, longitudinal) needs to determine the nature and direction of relationships. Please note that all factors are significantly influenced by age and gender, as discussed throughout the article, however, these are not outlined in detail here for ease of interpretation of the figure.

By further understanding the neurobiology of those who cyberbully and those who are cybervictims, appropriate treatment and interventions can be developed to address the short- and long-term effects of cyberbullying involvement. This review highlights that little research to date has addressed the relationship between cyberbullying and adolescent brain development. In particular, no research is yet to address the important role of age and gender during adolescent development, and how this may in turn influence how the brain is affected by cyberbullying. Despite the limitations in terms of the breadth and depth of the research conducted to date, we have developed a potential framework to depict the roles that the key factors we have addressed in this article may have in cyberbullying. Thus, Figure 1 summarizes how the changes in the brain and cognition occurring during adolescence may influence cyberbullying behaviors. More specifically, Figure 1 describes the key factors discussed in this article and how they relate to cyberbullying; the adolescent brain and mental health, which may be helpful in developing strategies and action plans for practitioners in cyberbullying prevention. Finally, the practical implications of this article and future research could inform educators on appropriately handling cyberbullying in schools and could guide clinicians on how to assist young people who are coping with cyberbullying experiences. Future researchers could also gain further insights around prevention and intervention research regarding cyberbullying. Moving forward, it is important to understand the influence that cyberbullying can have on the brain over time, especially as technology becomes more and more a part of adolescents (and adults) lives. As it stands, little is known about how the use of technology, particularly in relation to cyberbullying, is affecting the development of young people’s brains. In addition, cyberbullying education and interventions focus primarily on reducing cyberbullying in schools though means of restricting technology, or education around coping with cyberbullying. If research could elucidate the biological underpinning of cyberbullying, interventions could have a more targeted approach around *prevention*, as well as further understanding of the effect on young people developmentally. In addition, research such as this, especially longitudinal research, could identify those who may be at increased risk to developing mental health concerns as result of cyberbullying experiences, due to underlying neurobiological pre-dispositions.

## Author Contributions

LM was the lead author of this manuscript. JL and DH contributed to the content of this manuscript. All authors contributed to the article and approved the submitted version.

## Conflict of Interest

The authors declare that the research was conducted in the absence of any commercial or financial relationships that could be construed as a potential conflict of interest.

## References

[B1] Barnea-Goraly N. Menon V. Eckert M. Tamm L. Bammer R. Karchemskiy A. (2005). White matter development during childhood and adolescence: a cross-sectional diffusion tensor imaging study. *Cereb. Cortex* 15 1848–1854. 10.1093/cercor/bhi062 15758200

[B2] Blakemore S. J. Choudhury S. (2006). Development of the adolescent brain: implications for executive function and social cognition. *J. Child Psychol. Psychiatry* 47 296–312. 10.1111/j.1469-7610.2006.01611.x 16492261

[B3] Bradshaw C. P. Goldweber A. Fishbein D. Greenberg M. T. (2012). Infusing developmental neuroscience into school-based preventive interventions: implications and future directions. *J. Adolesc. Health* 51 S41–S47. 22794533 10.1016/j.jadohealth.2012.04.020 PMC13234207

[B4] Broadhouse K. M. Boyes A. Winks N. Dokonal T. Mcloughlin L. Parker M. (2019). Subcortical volume correlates of psychological distress in early adolescence. *Dev. Neurosci.* 41 193–202. 10.1159/000502339 31480044

[B5] Campbell M. Spears B. Slee P. Butler D. Kift S. (2012). Victims’ perceptions of traditional and cyberbullying, and the psychosocial correlates of their victimisation. *Emot. Behav. Diffic.* 17 389–401. 10.1080/13632752.2012.704316

[B6] Casey B. J. Jones R. M. Levita L. Libby V. Pattwell S. S. Ruberry E. J. (2010). The storm and stress of adolescence: insights from human imaging and mouse genetics. *Dev. Psychobiol.* 52 225–235. 20222060 10.1002/dev.20447 PMC2850961

[B7] Corrado R. Mathesius J. (2014). Developmental psycho- neurological research trends and their importance for reassessing key decision-making assumptions for children, adolescents, and young adults in juvenile/youth and adult criminal justice systems. *Bergen J. Law Crim. Justice* 2 141–163.

[B8] Crone E. A. Konijn E. A. (2018). Media use and brain development during adolescence. *Nat. Commun.* 9:588. 10.1038/s41467-018-03126-x PMC5821838 29467362

[B9] Cross D. Shaw T. Hearn L. Epstein M. Monks H. Lester L. (2009). *Australian Covert Bullying Prevalence Study (ACBPS).* Perth: Child Health Promotion Research Centre, Edith Cowan University.

[B10] Currie C. Zanotti C. Morgan A. Currie D. De Looze M. Roberts C. (2009). *Social Determinants of Health and Well-being Among Young People. Health Behaviour in School-aged Children (HBSC) Study: International Report from the 2010.* Copenhagen: WHO Regional Office for Europe, 271.

[B11] du Plessis M. R. Smeekens S. Cillessen A. H. N. Whittle S. Güroglu B. (2019). Bullying the brain? Longitudinal links between childhood peer victimization, cortisol, and adolescent brain structure. *Front. Psychol.* 9:2706. 10.3389/fpsyg.2018.02706 30692951 PMC6340095

[B12] Fahy A. E. Stansfeld S. A. Smuk M. Smith N. R. Cummins S. Clark C. (2016). Longitudinal associations between cyberbullying involvement and adolescent mental health. *J. Adolesc. Health* 59 502–509. 10.1016/j.jadohealth.2016.06.006 27528471

[B13] George M. J. Odgers C. L. (2015). Seven fears and the science of how mobile technologies may be influencing adolescents in the digital age. *Perspect. Psychol. Sci.* 10 832–851. 10.1177/1745691615596788 26581738 PMC4654691

[B14] Giedd J. N. (2008). The teen brain: insights from neuroimaging. *J. Adolesc. Health* 42 335–343. 10.1016/j.jadohealth.2008.01.007 18346658

[B15] Giedd J. N. Blumenthal J. Jeffries N. O. Castellanos F. X. Liu H. Zijdenbos A. (1999). Brain development during childhood and adolescence: a longitudinal MRI study. *Nat. Neurosci.* 2:861. 10.1038/13158 10491603

[B16] Giedd J. N. Snell J. W. Lange N. Rajapakse J. C. Casey B. J. Kozuch P. L. (1996). Quantitative magnetic resonance imaging of human brain development: ages 4–18. *Cereb. Cortex* 6 551–559. 10.1093/cercor/6.4.551 8670681

[B17] González-Cabrera J. Calvete E. León-Mejía A. Pérez-Sancho C. Peinado J. M. (2017). Relationship between cyberbullying roles, cortisol secretion and psychological stress. *Comp. Hum. Behav.* 70 153–160. 10.1016/j.chb.2016.12.054

[B18] Hankin B. L. Abramson L. Y. Moffitt T. E. Silva P. A. Mcgee R. Angell K. E. (1998). Development of depression from preadolescence to young adulthood: emerging gender differences in a 10-year longitudinal study. *J. Abnorm. Psychol.* 107:128. 10.1037/0021-843x.107.1.128 9505045

[B19] Hatton S. N. Lagopoulos J. Hermens D. F. Naismith S. L. Bennett M. R. Hickie I. B. (2012). Correlating anterior insula gray matter volume changes in young people with clinical and neurocognitive outcomes: an MRI study. *BMC Psychiatry* 12:45. 10.1186/1471-244X-12-45 22607202 PMC3468394

[B20] Hemphill S. A. Heerde J. A. (2014). Adolescent predictors of young adult cyberbullying perpetration and victimization among Australian youth. *J. Adolesc. Health* 55 580–587. 10.1016/j.jadohealth.2014.04.014 24939014 PMC4295930

[B21] Johnson S. B. Blum R. W. Giedd J. N. (2009). Adolescent maturity and the brain: the promise and pitfalls of neuroscience research in adolescent health policy. *J. Adolesc. Health* 45 216–221. 10.1016/j.jadohealth.2009.05.016 19699416 PMC2892678

[B22] Kim S. Georgiades K. Comeau J. Vitoroulis I. Boyle M. H. (2016). The association between cyberbullying victimization and adolescent mental health: a comparative study between traditional types of bullying versus cyberbullying. *J. Am. Acad. Child Adolesc. Psychiatry* 55:S160.

[B23] Lagopoulos J. Hermens D. F. Hatton S. N. Tobias-Webb J. Griffiths K. Naismith S. L. (2013). Microstructural white matter changes in the corpus callosum of young people with bipolar disorder: a diffusion tensor imaging study. *PLoS One* 8:e59108. 10.1371/journal.pone.0059108 23527101 PMC3602458

[B24] Lagopoulos J. Hermens D. F. Naismith S. L. Scott E. M. Hickie I. B. (2012). Frontal lobe changes occur early in the course of affective disorders in young people. *BMC Psychiatry* 12:4. 10.1186/1471-244X-12-4 22264318 PMC3280164

[B25] Lamblin M. Murawski C. Whittle S. Fornito A. (2017). Social connectedness, mental health and the adolescent brain. *Neurosci. Biobehav. Rev.* 80 57–68. 10.1016/j.neubiorev.2017.05.010 28506925

[B26] Le H. T. Nguyen H. T. Campbell M. A. Gatton M. L. Tran N. T. Dunne M. P. (2017). Longitudinal associations between bullying and mental health among adolescents in Vietnam. *Int. J. Public Health* 62 51–61. 10.1007/s00038-016-0915-8 27812723

[B27] Li Q. (2006). Cyberbullying in schools: a research of gender differences. *Sch. Psychol. Int.* 27 157–170. 10.1177/0143034306064547

[B28] Masten C. L. Eisenberger N. I. Borofsky L. A. Mcnealy K. Pfeifer J. H. Dapretto M. (2011). Subgenual anterior cingulate responses to peer rejection: a marker of adolescents’ risk for depression. *Dev. Psychopathol.* 23 283–292. 10.1017/s0954579410000799 21262054 PMC3229829

[B29] Masten C. L. Eisenberger N. I. Borofsky L. A. Pfeifer J. H. Mcnealy K. Mazziotta J. C. (2009). Neural correlates of social exclusion during adolescence: understanding the distress of peer rejection. *Soc. Cogn. Affect. Neurosci.* 4 143–157. 10.1093/scan/nsp007 19470528 PMC2686232

[B30] McLoughlin L. Spears B. Taddeo C. (2018). The importance of social connection for cybervictims: how connectedness and technology could promote mental health and wellbeing in young people. *Int. J. Emot. Educ.* 10 5–24.

[B31] McLoughlin L. T. Shan Z. Broadhouse K. M. Lagopoulos J. Winks N. Hermens D. F. (2020a). Elucidating the neurobiology of cyberbullying using functional magnetic resonance imaging(fMRI): a hypothesis. *Aggress. Violent Behav.* 50:101360 10.1016/j.avb.2019.101360

[B32] McLoughlin L. T. Shan Z. Broadhouse K. M. Winks N. Lagopoulos J. Hermens D. F. (2020b). Neurobiological underpinnings of cyberbullying: a pilot functional magnetic resonance imaging study. *Hum. Brain Mapp.* 41 1495–1504. 10.1002/hbm.24890 31797458 PMC7268014

[B33] McLoughlin L. T. Spears B. A. Taddeo C. M. Hermens D. F. (2019). Remaining connected in the face of cyberbullying: why social connectedness is important for mental health. *Psychol. Schools* 56 945–958. 10.1002/pits.22232

[B34] Mills K. L. Goddings A.-L. Herting M. M. Meuwese R. Blakemore S.-J. Crone E. A. (2016). Structural brain development between childhood and adulthood: convergence across four longitudinal samples. *Neuroimage* 141 273–281. 10.1016/j.neuroimage.2016.07.044 27453157 PMC5035135

[B35] Mills K. L. Lalonde F. Clasen L. S. Giedd J. N. Blakemore S.-J. (2014). Developmental changes in the structure of the social brain in late childhood and adolescence. *Soc. Cogn. Affect. Neurosci.* 9 123–131. 10.1093/scan/nss113 23051898 PMC3871734

[B36] Muetzel R. L. Mulder R. H. Lamballais S. Cortes Hidalgo A. P. Jansen P. Güroðlu B. (2019). Frequent bullying involvement and brain morphology in children. *Front. Psychiatry* 10:696. 10.3389/fpsyt.2019.00696 31607968 PMC6771170

[B37] Ostby Y. Tamnes C. K. Fjell A. M. Westlye L. T. Due-Tonnessen P. Walhovd K. B. (2009). Heterogeneity in subcortical brain development: a structural magnetic resonance imaging study of brain maturation from 8 to 30 years. *J. Neurosci.* 29 11772–11782. 10.1523/jneurosci.1242-09.2009 19776264 PMC6666647

[B38] Paus T. Keshavan M. Giedd J. N. (2008). Why do many psychiatric disorders emerge during adolescence? *Nat. Rev. Neurosci.* 9 947–957. 10.1038/nrn2513 19002191 PMC2762785

[B39] Pfefferbaum A. Mathalon D. H. Sullivan E. V. Rawles J. M. Zipursky R. B. Lim K. O. (1994). A quantitative magnetic resonance imaging study of changes in brain morphology from infancy to late adulthood. *Arch. Neurol.* 51 874–887. 10.1001/archneur.1994.00540210046012 8080387

[B40] Quinlan E. B. Barker E. D. Luo Q. Banaschewski T. Bokde A. L. W. Bromberg U. (2018). Peer victimization and its impact on adolescent brain development and psychopathology. *Mol. Psychiatry* 1–11. 10.1038/s41380-018-0297-9 30542059

[B41] Reiss A. L. Abrams M. T. Singer H. S. Ross J. L. Denckla M. B. (1996). Brain development, gender and IQ in childrenA volumetric imaging study. *Brain* 119 1763–1774. 10.1093/brain/119.5.1763 8931596

[B42] Sakellariou T. Carroll A. Houghton S. (2012). Rates of cyber victimization and bullying among male Australian primary and high school students. *Sch. Psychol. Int.* 33 533–549. 10.1177/0143034311430374

[B43] Sherman L. E. Payton A. A. Hernandez L. M. Greenfield P. M. Dapretto M. (2016). The power of the like in adolescence: effects of peer influence on neural and behavioral responses to social media. *Psychol. Sci.* 27 1027–1035. 10.1177/0956797616645673 27247125 PMC5387999

[B44] Smith P. K. Jones A. P. (2012). The importance of developmental science for studies in bullying and victimization. *Int. J. Dev. Sci.* 6 71–74. 10.3233/dev-2012-11093

[B45] Smith P. K. Mahdavi J. Carvalho M. Fisher S. Russell S. Tippett N. (2008). Cyberbullying: its nature and impact in secondary school pupils. *J. Child Psychol. Psychiatry* 49 376–385. 10.1111/j.1469-7610.2007.01846.x 18363945

[B46] Sowell E. R. Peterson B. S. Thompson P. M. Welcome S. E. Henkenius A. L. Toga A. W. (2003). Mapping cortical change across the human life span. *Nat. Neurosci.* 6 309–315. 10.1038/nn1008 12548289

[B47] Sowell E. R. Thompson P. M. Tessner K. D. Toga A. W. (2001). Mapping continued brain growth and gray matter density reduction in dorsal frontal cortex: Inverse relationships during postadolescent brain maturation. *J. Neurosci.* 21 8819–8829. 10.1523/jneurosci.21-22-08819.2001 11698594 PMC6762261

[B48] Spears B. A. Taddeo C. M. Daly A. L. Stretton A. Karklins L. T. (2015). Cyberbullying, help-seeking and mental health in young Australians: implications for public health. *Int. J. Public Health* 60 219–226. 10.1007/s00038-014-0642-y 25572385

[B49] Steinberg L. (2005). Cognitive and affective development in adolescence. *Trends Cogn. Sci.* 9 69–74. 10.1016/j.tics.2004.12.005 15668099

[B50] Steinberg L. (2013). The influence of neuroscience on US Supreme Court decisions about adolescents' criminal culpability. *Nat. Rev. Neurosci.* 14:513. 10.1038/nrn3509 23756633

[B51] Vaillancourt T. Hymel S. Mcdougall P. (2013). The biological underpinnings of peer victimization: understanding why and how the effects of bullying can last a lifetime. *Theory Into Pract.* 52 241–248. 10.1080/00405841.2013.829726

[B52] van Geel M. Vedder P. Tanilon J. (2014). Relationship between peer victimization, cyberbullying, and suicide in children and adolescents: a meta-analysis. *JAMA Pediatr.* 168 435–442. 24615300 10.1001/jamapediatrics.2013.4143

[B53] Zych I. Ortega-Ruiz R. Del Rey R. (2015). Systematic review of theoretical studies on bullying and cyberbullying: facts, knowledge, prevention, and intervention. *Aggress. Violent Behav.* 23 1–21. 10.1016/j.avb.2015.10.001

